# Ten Color Multiparameter Flow Cytometry in Bone Marrow and Apheresis Products for Assessment and Outcome Prediction in Multiple Myeloma Patients

**DOI:** 10.3389/fonc.2021.708231

**Published:** 2021-08-13

**Authors:** Veronika Riebl, Sandra Maria Dold, Dagmar Wider, Marie Follo, Gabriele Ihorst, Johannes M. Waldschmidt, Johannes Jung, Michael Rassner, Christine Greil, Ralph Wäsch, Monika Engelhardt

**Affiliations:** ^1^Department of Medicine I Hematology and Oncology, Faculty of Medicine, Medical Center – University of Freiburg, Freiburg, Germany; ^2^Faculty of Biology, University of Freiburg, Freiburg, Germany; ^3^Clinical Trials Unit, Faculty of Medicine, Medical Center – University of Freiburg, Freiburg, Germany; ^4^Comprehensive Cancer Center Freiburg (CCCF), Faculty of Medicine, Medical Center – University of Freiburg, Freiburg, Germany

**Keywords:** minimal residual disease, multiple myeloma, multiparameter flow cytometry, improved progression-free survival, phenotypic analyses, bone marrow, apheresis product

## Abstract

**Objective:**

In clinical trials (CTs), the assessment of minimal residual disease (MRD) has proven to have prognostic value for multiple myeloma (MM) patients. Multiparameter flow cytometry (MFC) and next-generation sequencing are currently used in CTs as effective tools for outcome prediction. We have previously described 6- and 8-color MFC panels with and without kappa/lambda, which were equally reliable in detecting aberrant plasma cells (aPC) in myeloma bone marrow (BM) specimens. This follow-up study a) established a highly sensitive single-tube 10-color MFC panel for MRD detection in myeloma samples carrying different disease burden (monoclonal gammopathy of unknown significance (MGUS), smoldering multiple myeloma (SMM), MM), b) evaluated additional, rarely used markers included in this panel, and c) assessed MRD levels and the predictive value in apheresis vs. BM samples of MM patients undergoing autologous stem cell transplantation (ASCT).

**Methods + Results:**

The 10-color MFC was performed in BM and apheresis samples of 128 MM and pre-MM (MGUS/SMM) patients. The markers CD28, CD200, CD19, and CD117 underwent closer examination. The analysis revealed distinct differences in these antigens between MM, MGUS/SMM, and patients under treatment. In apheresis samples, the 10-color panel determined MRD negativity in 44% of patients. Absence of aPC in apheresis corresponded with disease burden, cytogenetics, and response to induction. It also determined MRD negativity in BM samples after ASCT and was associated with improved progression-free survival.

**Conclusion:**

These results highlight the significance of the evaluation of both BM and apheresis samples with a novel highly sensitive 10-color MFC panel.

## Introduction

Multiple myeloma (MM) is characterized by the accumulation of aberrant plasma cells (aPC) in the bone marrow (BM). More recently, peripheral blood liquid biopsies and medical imaging have garnered significant interest in the scientific community for their potential to serially assess MM disease burden (1, 2). BM remains the most sensitive source for minimal residual disease (MRD) detection to date and has been the standard in numerous investigations (3). Nevertheless, significant advances have been made in the development of assays that could provide further insight into the disease heterogeneity outside of osteolytic sites (4–6). For MRD testing, both multiparameter flow cytometry (MFC) and next-generation sequencing (NGS), with sensitivity thresholds of 10^-4^ to 10^-6^, are used in clinical trials as effective and adaptable tools for the early prediction of overall response rates (ORR), progression-free survival (PFS), and overall survival (OS) (7). While NGS has the advantage of better performance on limited cell numbers, MFC provides results within hours, does not require a baseline sample, and is applicable to nearly 100% of patients (8). The establishment of this method in MRD detection has largely been advanced by the EuroFlow consortium (9). However, with special equipment requirements, cost, and time limitations, MFC panels are not routinely available for all MM patients in- or outside clinical trials. Whether 6-, 8-, or 10-color MFC assays are most practical, sensitive, and valid has rarely been tested side-by-side. In a recent extensive analysis by our group, we had first established a 6-color panel composed of the antigens CD138, CD38, CD19, CD45, CD27, and CD56 (10). The hereby acquired data suggested a potential for improvement in aberrant plasma cell (aPC) detection. Thus, we conceptualized an 8-color panel (consisting of the 6-color panel plus kappa and lambda) and this 10-color panel simultaneously to answer a variety of different research questions. The comparison of the 6-color panel and the 8-color panel has already been published and showed that both panels with and without kappa/lambda were equally robust and reliably detected aPC and normal plasma cells (nPC), with kappa/lambda being an additional tool for assessing clonality (10). Thus, in this paper, we focused on the evaluation of potential benefits and disadvantages of the 10-color panel.

Here, we assessed the utility and sensitivity of the 10-color panel in MM and pre-MM [monoclonal gammopathy of unknown significance (MGUS), smoldering multiple myeloma (SMM)] patient samples, treated both within and outside of clinical trials regarding aPC vs. nPC detection.

We investigated the effectiveness of additional markers (i.e., CD200, CD81, CD28, and CD117) at pre-MM stage (MGUS/SMM), at initial diagnosis of MM (ID), during disease progression (PD), under anti-MM treatment, and whether they could provide a similar or increased reliability in identification of aPC compared to kappa/lambda. These markers had been reported to either have a high expression in MM cells, have predictive potential, or had not been included in commercially available panels like EuroFlow or from the Memorial Sloan Kettering Cancer Center (MSKCC) (5, 7).

Furthermore, as PB vs. BM has been tested likewise, we wanted to explore whether MRD-detection in apheresis products from patients who underwent autologous stem cell transplantation (ASCT) was feasible and had implications for PFS.

## Methods

A total volume of 2 ml of either BM aspirates or apheresis samples was lysed and directly stained with the cell-surface antibodies CD138(APC), CD38(PE-Cy7), CD45(APC-H7), CD56(PerCp-Cy5.5), CD27(PE), CD19(BV510), CD81(FITC), CD200(BV421), CD117(BV786), and CD28(BV605) (**Table 1**). For all analyses, 3 × 10^6^ events were acquired on a BD LSR Fortessa™ flow cytometer. Patients receiving daratumumab treatment were excluded from the study, as this may interfere with CD38 detection.

**Table 1:** Key data for the ten investigated antigens and utilized fluorochromes.

Antigen	Clone	Fluorescence	Manufacturer	Aberrant expression	Rationale for selection
CD138	MI15	APC	Becton Dickinson	++	Standard PC/MM identification
CD38	HB7	PE-Cy7	Becton Dickinson	++	Standard PC/MM identification
CD27	1A4CD27	PE	BeckmanCoulter	-/+	Included in most MM panels and the 6-color panel
CD19	SJ25C1	BV510	Becton Dickinson	-/+	Included in most MM panels and the 6-color panel
CD56	B159	PerCP-Cy5.5	Becton Dickinson	+/-	Included in most MM panels and the 6-color panel
CD45	2D1	APC-H7	Becton Dickinson	–	Included in most MM panels and the 6-color panel
CD81	JS64	FITC	BeckmanCoulter	-/+	Association with t(11;14)
CD117	104D2	BV786	Becton Dickinson	++	No positive nPC expression reported, strictly positive in MM
CD28	CD28.2	BV605	Becton Dickinson	+++	Highly positive in MM; not included in EuroFlow
CD200	OX-104	BV421	Becton Dickinson	+++	Highly positive in MM; not included in EuroFlow

Galtseva et al. Int J Lab Hem 2017, Sarasquete et al. Haematologica 2015, Silvennoinen et al. Nature BCJ 2014, Rawstron et al. Blood 2015, Paiva et al. Haematologica 2015, Paiva et al. Clinical cancer research 2015, Rawstron et al. (11).

Aberrant phenotypes for the investigated markers were defined as CD138^+^CD38^+^CD56^+/-^CD45^-^CD19^-/+^CD27^-/+^CD117^+^CD200^+^CD28^+^CD81^-^.

Normal phenotypes were defined as CD138^+^CD38^+^CD56^-/+^CD45^+^CD19^+/-^CD27^+^CD117^-^CD200^-^CD28^-^CD81^+^. Samples were termed MRD negative (MRD^-^) with detection of <10^-5^ aPC of total nucleated cells. Validation analyses were performed as previously described (10). The limit of detection (LOD) was determined *via* independent dilution assays and resulted in a high sensitivity of 10^-5^.

Written consent was obtained from all patients, and the study was approved by the local ethics committee. Statistical analysis was performed using the Mann-Whitney U test for unpaired samples, Wilcoxon signed-rank test for paired samples, and the log-rank test for survival curves. For all analyses, comparisons were considered statistically significant for p-values <0.05.

## Results

### Aberrant and Normal PC in Myeloma *vs*. Precursor Disease (MGUS/SMM) Bone Marrow, in Apheresis Samples and in Healthy Donor BM Samples

**Figure 1:**
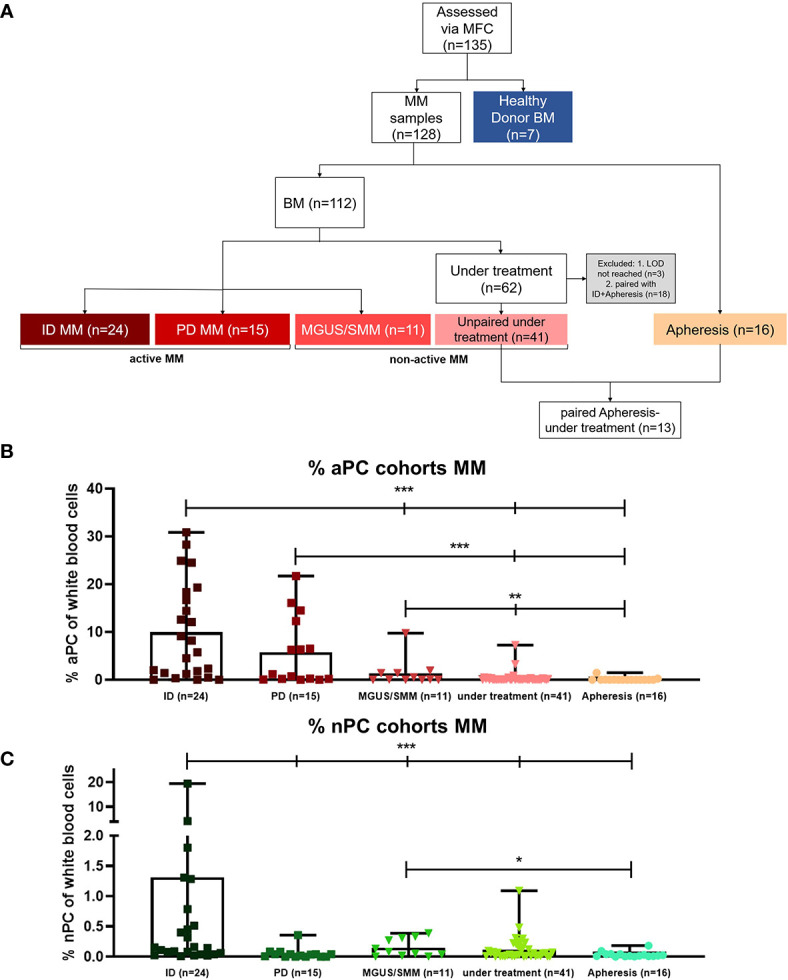
Analyzed cohorts: patient sample flow diagram and percentage of aberrant plasma cells (aPC) and normal plasma cells (nPC) of different MM cohorts. **(A)** Flow diagram of the analyzed patient cohorts. A total number of 135 samples was analyzed. Apheresis (n = 16) and bone marrow (BM) (n = 112) samples of multiple myeloma (MM) patients were measured. Out of 112 BM samples, 24 were from patients presenting at initial diagnosis (ID), 15 from patients with progressive disease (PD), 11 from patients with monoclonal gammopathy of unknown significance (MGUS) or smoldering multiple myeloma (SMM), and 62 from patients under treatment. Three samples from patients under treatment were excluded because they did not reach the limit of detection (LOD). For the cohort comparisons, 18 patient samples had to be excluded because they were paired with patients in other cohorts, bringing the number to 41 unpaired under treatment samples. Out of those 18 paired samples under treatment, 13 samples were paired follow-up samples of patients that had been previously assessed at the time of stem cell harvest in apheresis products. For mean fluorescence intensity (MFI) analysis, some patients showed either aPC or nPC populations that were below the LOD. Seven BM samples of healthy donors (HD) were examined. **(B)** Comparison of aPC percentages of total nucleated BM cells in the investigated MM cohorts (p < 0.0070; p < 0.0001; Mann-Whitney U test). **(C)** Comparison of nPC percentages of total nucleated BM cells in the investigated MM cohorts (p < 0.0001; Mann-Whitney U test). ***p < 0.0001; **p < 0.001; *p < 0.01.

A total of 135 samples was analyzed (**Figure 1A**), including 128 pre-MM/MM and 7 HD BM samples. In the MM group, 112 BM and 16 apheresis samples were assessed. BM samples consisted of patients with active disease [defined as ID (n = 24) or PD (n = 15)] vs. non-active MM [defined as MGUS/SMM (n = 11) or under anti-MM treatment (n = 41)]. The assay was applicable to 96% of all samples, and reasons for sample exclusion are given in **Figure 1A** [for mean fluorescence intensity (MFI) analysis, some samples were below the limit of detection (LOD)].

We first analyzed differences in relative frequencies of aPC and normal plasma cells (nPC) in the BM between different groups of MM patients as depicted in **Figures 1B, C**, respectively. As expected, the frequency of aPC in the BM was much higher in patients with active MM disease, such as ID or PD patients, in comparison to those with MGUS/SMM, MM patients under treatment, or in the apheresis collections (p < 0.0001; **Figure 1B**).

nPC in different MM samples (**Figure 1C**) showed much lower frequencies than aPC (**Figure 1B**). nPC from ID BM samples was higher than in all other subgroups, including MGUS/SMM and apheresis samples (p < 0.0001). In five ID patient samples, the 10-color panel identified higher nPC percentages than expected, suggesting a potential utility of kappa/lambda staining in those cases.

The combined analysis of samples from patients with active vs. non-active MM, for both aPC and nPC, is depicted in **Supplementary Figure S1A–C**. It confirmed significantly higher levels of aPC in active MM (**A**), similar percentages of nPC (**B**), and substantial differences of aPC vs. nPC in active MM samples (**C**).

### Evaluation of Each Antigen Marker Individually *via* 10-Color MFC Assay

**Figure 2:**
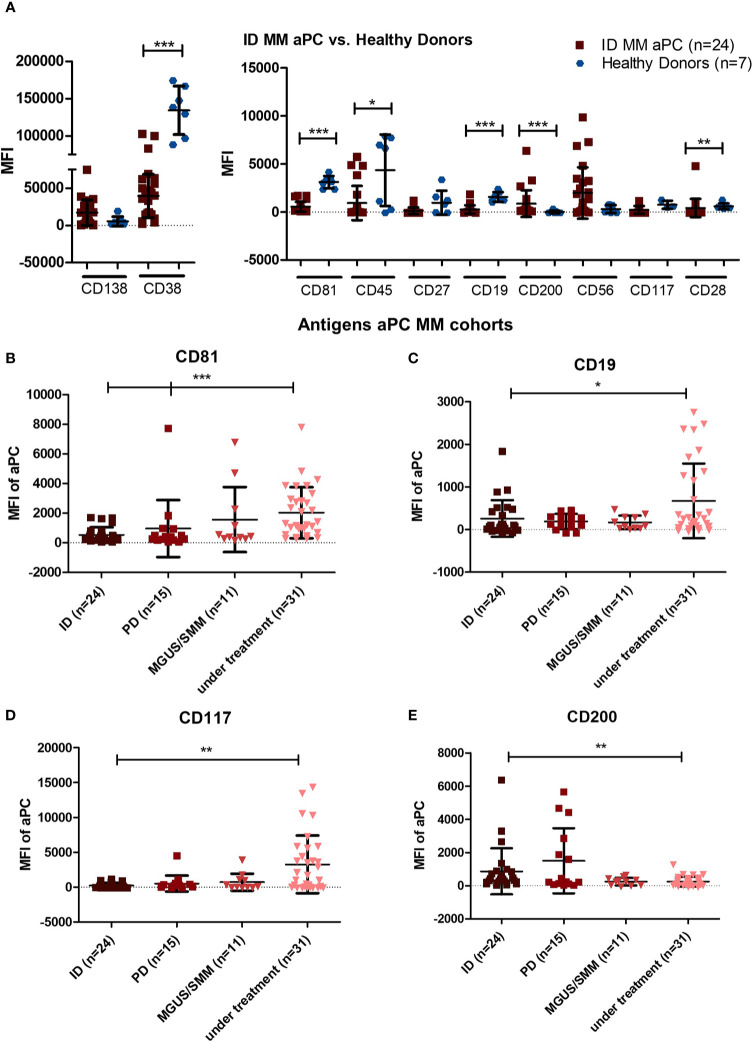
Phenotypic analysis of the 10 antigens on aPC in BM of MM patients and healthy donors. **(A)** Geometric mean fluorescence intensity (MFI) analysis of healthy donor samples compared to aPC of ID MM patients (p < 0.0001; Mann-Whitney U test). Due to technical errors, four healthy donor samples had to be excluded for CD117 MFI analysis. **(B)** MFI analysis of CD81 solely on aPC for the investigated cohorts (p < 0.0001; p = 0.0004; Mann-Whitney test). **(C)** MFI analysis of CD19 solely on aPC for the investigated cohorts (p = 0.0469; Mann-Whitney U test). **(D)** MFI analysis of CD117 solely on aPC for the investigated cohorts (p = 0.0019; Mann-Whitney U test). **(E)** MFI analysis of CD200 solely on aPC for the investigated cohorts (p = 0.0056; Mann-Whitney U test). ***p < 0.0001; **p < 0.001; *p < 0.01.

To evaluate the usefulness and predictive value of each antigen marker individually in our 10-color MFC assay, MFI values of aPC in ID MM samples were compared with HD BM samples (**Figure 2A**). MFI showed highly significant expression differences for CD38, CD81, CD19, and CD200 (p < 0.0001). Due to the phenotypical diversity of MM cells, markers such as CD27 and CD56 had a wider range of expression across the samples; thus, not every marker showed statistically significant differences to HD samples.

To determine potential differences of MFI in MM samples at ID, PD, in MGUS/SMM, and those under treatment, four markers were assessed separately in aPC (**Figures 2B–E**). CD81, CD19, and CD200 showed the most significant differences for aPC in ID MM samples as compared to HD samples. In a more extensive analysis of markers in under treatment samples, a noteworthy dynamic for CD117 MFI presented itself as well. Thus, CD81, CD19, CD117, and CD200 were selected for independent MFI investigation. Of interest, CD81, CD19, and CD117 were increased in ID, PD, MGUS/SMM, and MM samples under treatment, being highest in the latter subgroup, while CD200 showed a decrease (**Figures 2B–E**).

Differences of nPC to HD BM and in the different patient cohorts are depicted in **Supplementary Figures S2A–E**, where most significant MFI variations were seen for CD38 (p < 0.0001; **Supplementary Figures S2A**) and no changes were seen for CD81, CD19, CD117, and CD200 in the different cohorts (**Supplementary Figures S2B–E**).

Differences in the four markers CD81, CD19, CD117, and CD200 were also assessed for active (ID + PD) vs. non-active (MGUS/SMM + under treatment) BM samples, distinguishing both aPC (**Supplementary Figure S3A–D**) and nPC (**Supplementary Figure S3E–H**). Decreased MFI was apparent for CD81, CD19, and CD117 for active MM samples, whereas for CD200 the MFI was increased in active compared to non-active MM samples.

Marker expression of CD81, CD19, CD117, and CD200 in nPC was not significantly different in samples of patients with active vs. non-active disease (**Supplementary Figure S3E–H**).

### MFC Utilized in Apheresis Samples of MM Patients

**Figure 3:**
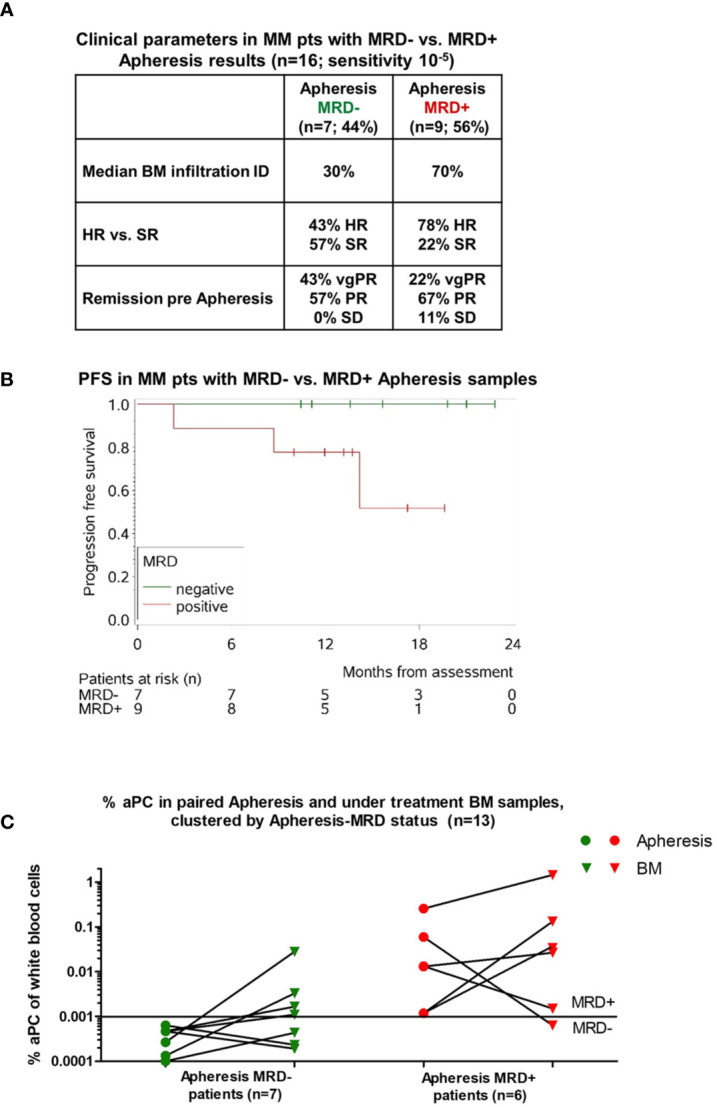
Analysis of apheresis samples. **(A)** Clinical parameters of MM patients with MRD^-^ vs. MRD^+^ apheresis product results. Remission pre-apheresis according to International Myeloma Working Group (IMWG) criteria, median BM infiltration at ID, and cytogenetics (high risk vs. standard risk) of 16 patients assessed at stem cell harvest were compared based on MRD status in apheresis samples (MRD^-^ n = 7; MRD^+^ n = 9; Sensitivity 10^-5^). **(B)** The Kaplan-Meier curve shows the progression-free survival (PFS) of patients with MRD^-^ apheresis samples compared to MRD^+^ apheresis samples (p = 0.12; log-rank test). **(C)** Percentage of aPC in paired apheresis and under treatment BM samples, clustered by apheresis-MRD status (n = 13).

Based on these BM results, we then investigated whether this MFC panel could be utilized in apheresis samples of MM patients having received three cycles of bortezomib-cyclophosphamide-dexamethasone (VCD) induction, cyclophosphamide-etoposide (CE) mobilization, and scheduled ASCT. MRD status was assessed in 16 apheresis samples: 7 patients reached aPC levels below a sensitivity of <10^-5^ (MRD^-^, **Figure 3A**). Compared to MRD positive (MRD^+^) apheresis samples (n = 9), MRD^-^ apheresis samples had lower BM infiltration rates at ID (30% vs. 70%), displayed high-risk (HR) cytogenetics less frequently (43% vs. 78%), and patients obtained better remission at apheresis time points (very good partial remission (vgPR) in 43% vs. 22%, respectively; **Figure 3A**).

PFS of patients with MRD^-^ vs. MRD^+^ apheresis samples showed distinct differences, with no disease progression in any of the seven MRD^-^ patients during our follow-up (FU) period (median FU: 15 months; range: 9–22), while 3/9 MRD^+^ patients showed disease progression (**Figure 3B**). Due to the limited number of apheresis samples, this failed to reach significance (p = 0.12; log-rank test). Similar to our previous extensive analysis (10), OS differences were not assessable as no events occurred within the observation period.

Since BM samples are routinely obtained at ID and after ASCT at our institution to determine remission post ASCT, additional matched BM samples at the time of apheresis assessment were not available, but rather at a time approximately 1 month later. This allowed us to determine whether prior MRD^-^ vs. MRD^+^ apheresis samples matched with later BM MRD results after ASCT. Out of the 16 apheresis samples examined, 13 patients had paired BM samples at a median of 40 days after ASCT. Of these matched apheresis/BM samples, seven patients were MRD^-^ and six patients were MRD^+^ in their respective apheresis sample (**Figure 3C**). Out of seven patients with MRD^-^ apheresis samples, three also revealed MRD^-^ status within the BM, while four of seven were MRD^+^ post-ASCT. Of the six MRD^+^ apheresis samples, all except one were also MRD^+^ in paired post-ASCT BM samples (**Figure 3C**).

### Comparison of the 10-Color Panel With Our 8-Color Panel Including Kappa/Lambda

**Figure 4:**
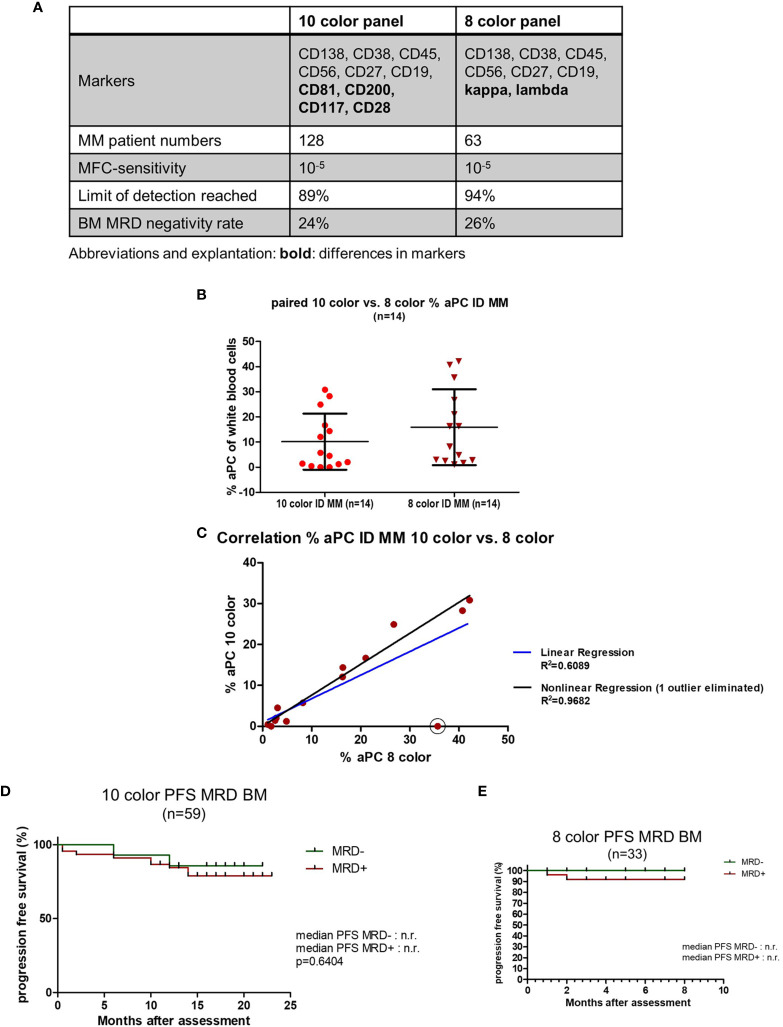
Comparison with 8-color MFC panel including kappa/lambda. **(A)** Comparison of the 10-color panel with the previously established 8-color panel including kappa/lambda. Markers, number of assessed samples, sensitivity, percentage of samples that reached the limit of detection (LOD), and BM negativity rate were compared. **(B)** Comparison of aPC percentage in paired 10-color and 8-color ID MM samples (n = 14). **(C)** Correlation analysis of aPC percentage measured *via* the 10-color and 8-color panel in 14 paired ID MM BM samples. Linear regression in blue (R^2^ = 0.6089) and nonlinear regression eliminating one outlier (circled) in black (R^2^ = 0.9682). **(D)** Kaplan-Meier analysis of progression-free survival in the 10-color BM MRD cohort for MRD- vs. MRD+ patients (n = 59). **(E)** Kaplan-Meier analysis of progression-free survival in the 8-color BM MRD cohort for MRD- vs. MRD+ patients (n = 33).

To further validate our panel and assess the necessity of kappa/lambda staining, we conducted a comparison of our 10-color panel and the previously published 8-color panel (10). This comparison was performed in similar, but not identical patient cohorts, as the 8-color panel was only assessed in 63 patient samples (compared to 128 for the 10-color panel). As shown in **Figure 4A**, the 8- and 10-color panels were comparable concerning sensitivity (10^-5^ for both) and consistency in sensitivity (LOD was reached in 89% and 96% of MRD samples, respectively). While a total of 24 ID MM samples were assessed using the 10-color panel, 14 of those samples were also measured using the 8-color panel (**Figure 4B**). Out of those 14 paired samples, only 1 sample showed a discrepancy in aPC infiltration measured by the 10-color and 8-color panel (1.5% vs. 35% aPC of white blood cells). This was due to a strictly normal expression of every 10-color gating marker in the single predominant subpopulation, and thus, aPC was only detectable *via* kappa/lambda. The correlation analysis showed that with this outlier eliminated, the panels correlated strikingly well (R^2^ = 0.9682; **Figure 4C**). In terms of MRD comparison, we observed similar PFS Kaplan-Meier results and MRD negativity rates (24% vs. 26%) in MM patients, suggesting the validity of this 10-color panel for MRD assessment (**Figures 4D, E**).

## Discussion

In the present study, we demonstrate that our 10-color single-tube MFC assay proved applicable in 96% of distinct samples, showed highly reliable results, and was consistent with previous studies in detecting aPC and nPC in myeloma BM and apheresis samples (7). Using a single-tube assay minimizes the laborious workflow, without requiring special equipment.

Our panel incorporated antigens rarely included in myeloma MRD panels, like CD200 and CD28. CD28, in contrast to CD200, did not show significant differences between myeloma cohorts, but was increased in ID (**Figure 2A**), thus allowing the distinction between aPC and nPC (**Figure 2A** and **Supplementary Figure S2**). Out of the four additional markers, CD81 and CD200 showed, in the majority of patients, the clearest distinction between aPC and nPC and proved to be beneficial for MRD assessment. CD117 and CD28 were also distinctive in aPC/nPC distinction; however, only a subgroup of patients expressed these markers. Thus, aPC detection was less reliable.

With the development of therapeutic antibodies such as daratumumab, isatuximab, or elotuzumab, MFI analysis of different antigens may play an important role in future MM treatment decisions (12). We were able to show that there are differences in MFI phenotypes between MM samples, precursor diseases (MGUS/SMM), and patients under treatment, primarily in aPC antigen expression rather than in nPC (**Figures 1** and **2**, **Supplementary Figure S3**). Our analysis was deliberately performed in different PC dyscrasia patients with distinctive disease stages. In comparison, Arana et al. performed phenotypic analyses in patients throughout their disease course and observed that CD81 increased between ID and after treatment initiation (13), which corresponds with our findings (**Figure 2B**). As MFC results reflect the expression of surface antigens, it remains to be seen as to whether these changes can also be retraced at the genetic level (14). In addition, identifying the drivers of these phenotypic changes and the potential influence of anti-MM agents may also yield relevant results. For example, CD200 negativity has been shown to respond to combined treatment with proteasome inhibitor and immunomodulatory drugs with longer OS compared to conventional high-dose chemotherapy (15). Prior studies on the clinical relevance and predictive value of various markers in our panel have been conducted. However, due to conflicting findings regarding prognosis concerning CD200 and CD117, their predictive value is not yet clear (16, 17). Therefore, survival studies with our panel and these markers are of future interest.

Furthermore, MRD negativity as determined by our panel in apheresis samples was correlated with lower disease burden, more favorable cytogenetics, improved responses to induction treatment (**Figure 3A**) and PFS (**Figure 3B**), and determined MRD negativity in BM samples after ASCT (**Figure 3C**).

In paired apheresis and BM samples before and after ASCT, MRD negativity was likely to translate into MRD negativity in BM samples after ASCT, whereas in MRD^+^ apheresis samples, all except one remained MRD^+^ in post-ASCT BM samples. The MRD^+^ BM samples after ASCT in patients who achieved MRD negativity in apheresis samples may therefore reflect the residual MM disease within the patient, and thus, the difference confirms the significance of assessing various sites (i.e., in apheresis and BM). Previous investigations of apheresis samples of MM patients have been reported to predict PFS, albeit using limited MFC panels and without distinction between aPC and nPC (18). Those studies focusing on the occurrence of aPC in apheresis samples reported different levels of contamination (23% vs. 48% of patients) (19, 20). Our 10-color panel uncovered an occurrence of aPC in 56% of patients. Except for one, those patients also showed MRD positivity in their post-ASCT BM samples. Thus, our data highlights the significance of the sensitive evaluation of both BM and apheresis samples. Taking only 2 ml samples of apheresis products is neither invasive nor logistically difficult, and as previous investigations using PET imaging have uncovered sampling bias with BM aspirates alone (14), assessing apheresis *via* MFC may present a relevant additional tool for patients’ individual risk stratification, therapeutic decision-making, and further optimization of MM patient care. Furthermore, taking into consideration that disease evolution is particularly relevant in MM patients, regular risk assessment throughout the course of disease has been demonstrated to provide a more reliable conditional survival estimation than assessment at ID alone (21). Accordingly, in the present study, MRD status was assessed at multiple time points, thus enabling improved risk stratification for the corresponding patients.

We also compared this 10-color panel with our previously published 8-color panel [**Figure 4**; (10)]. While sensitivity, reliability, and robustness of the different panels were at similar levels, inclusion of kappa/lambda staining into this MFC panel seemed beneficial, as in select cases it allows for better identification of light-chain restricted clones (10). A disadvantage of the 8-color panel was the prolonged staining procedure. Moving forward, a combination panel composed of the 8-color panel plus CD200 and CD81 would provide a valid and robust assay for future MRD studies.

In this study, we show that a) our single-tube 10-color MFC panel was reliable for MRD detection in the whole spectrum of monoclonal gammopathies (MGUS, SMM, and MM) with a consistently high sensitivity, b) the additional markers included in this panel, prominently CD81 and CD200, were valuable and informative for MRD detection, while in select cases kappa/lambda proved beneficial, and c) MRD assessment was possible in apheresis samples likewise to BM, with MRD apheresis status holding predictive value for responses after ASCT.

In summary, our highly sensitive single-tube 10-color MFC panel provides reliable results in a wide range of BM samples, contextualizes previous findings in MM antigen expression, and supports the rationale for apheresis product assessment. Our study is limited by the single-institution design, no side-by-side comparison with commercially available MRD-Flow panels and limited sample size. Nevertheless, based on this study, further investigation of apheresis products and clinical trials seem warranted.

## Data Availability Statement

The raw data supporting the conclusions of this article will be made available by the authors, without undue reservation.

## Ethics Statement

The studies involving human participants were reviewed and approved by Ethikkommission der Albert-Ludwig-Universität Freiburg. The patients/participants provided their written informed consent to participate in this study.

## Author Contributions

VR analyzed the data. VR and ME wrote the manuscript. SD, VR, DW, RW, and ME designed the project. VR, SD, and DW measured the samples. GI contributed to the statistical analyses. MF provided insights into flow cytometry and provided equipment. JJ, JW, DW, RW, SD, MR, MF, CG, and GI revised the manuscript. RW and ME supported the project. All authors contributed to the article and approved the submitted version.

## Funding

The work was supported through Deutsche Krebshilfe grants #109569 (to ME, RW) and #111424 (to ME, RW). The Ethics number was 212/16.

## Conflict of Interest

RW has received research and travel support from Sanofi, Gilead, Jazz, Celgene, and Amgen, and has received consultancy fees from Sanofi, Pfizer, Gilead, Novartis, Amgen, and Takeda. ME has received educational and trial support from Amgen, Celgene, Takeda, BMS, Janssen, Novartis, and Karyopharm, and has received honoraria and consultancy fees from BMS, Celgene, Amgen, Takeda, Novartis, and Janssen, all unrelated to this study.

The remaining authors declare that the research was conducted in the absence of any commercial or financial relationships that could be construed as a potential conflict of interest.

## Publisher’s Note

All claims expressed in this article are solely those of the authors and do not necessarily represent those of their affiliated organizations, or those of the publisher, the editors and the reviewers. Any product that may be evaluated in this article, or claim that may be made by its manufacturer, is not guaranteed or endorsed by the publisher.
