# Supplementary material for: Ten Color Multiparameter Flow Cytometry in Bone Marrow and Apheresis Products for Assessment and Outcome Prediction in Multiple Myeloma Patients
Source: Front Oncol. 2021 Aug 13;11:708231. doi: 10.3389/fonc.2021.708231 (PMC8414971; doi:10.3389/fonc.2021.708231)
Supplement: Supplementary file 1 [file DataSheet_1.zip › Supplementary material.pdf]

Supplementary

Supplementary Material

Antigen	Clone	Fluorescence	Manufacturer
CD138	MI15	APC	Becton Dickinson
CD38	HB7	PE-Cy7	Becton Dickinson
CD27	1A4CD27	PE	BeckmanCoulter
CD19	SJ25C1	BV510	Becton Dickinson
CD56	B159	PerCP-Cy5.5	Becton Dickinson
CD45	2D1	APC-H7	Becton Dickinson
CD81	JS64	FITC	BeckmanCoulter
CD117	104D2	BV786	Becton Dickinson
CD28	CD28.2	BV605	Becton Dickinson,
CD200	OX-104	BV421	Becton Dickinson

Supplementary Data

Supplementary Figure 1. Percentage of aPC and nPC of MM patients with active vs. non-active disease
A Comparison of aPC percentages of total nucleated BM cells from patients with active and non-active MM ($p<0.0001$; Mann-Whitney U test).
B Comparison of nPC percentages of total nucleated BM cells from patients with active and non-active MM.
C Comparison of aPC percentages and nPC percentages of total nucleated BM cells from patients with active MM ($p<0.0001$; Mann-Whitney U test).

Supplementary Figure 2. Phenotypic analysis of the 10 antigens on nPC in BM of MM patients and healthy donors
A MFI values of the 10 investigated antigens in healthy donor samples were compared to MFI values on nPC of ID MM patients (Mann-Whitney U test). CD200, CD117 and CD28 are negative for nPC and HD and are thus included only for the sake of completeness.
B Comparison of CD81 MFI solely on nPC for the investigated MM cohorts (Mann-Whitney U test).
C Comparison of CD19 MFI solely on nPC for the investigated MM cohorts (Mann-Whitney U test).
D Comparison of CD117 MFI solely on nPC for the investigated MM cohorts (Mann-Whitney U test).
E Comparison of CD200 MFI solely on nPC for the investigated MM cohorts (Mann-Whitney U test).

Supplementary Figure 3. Phenotypic analysis of the 10 antigens in BM from patients with active vs. non-active MM
A MFI analysis of CD81 solely on aPC for active vs. non-active MM ($p<0.0001$; Mann-Whitney test).
B MFI analysis of CD19 solely on aPC for active vs. non-active MM.
C MFI analysis of CD117 solely on aPC for active vs. non-active MM ($p=0.0072$; Mann-Whitney test).
D MFI analysis of CD200 solely on aPC for active vs. non-active MM ($p=0.0027$; Mann-Whitney test).
E MFI analysis of CD81 solely on nPC for active vs. non-active MM.
F MFI analysis of CD19 solely on nPC for active vs. non-active MM.
G MFI analysis of CD117 solely on nPC for active vs. non-active MM.
H MFI analysis of CD200 solely on nPC for active vs. non-active MM.
