# Supplementary figures and images for: Ten Color Multiparameter Flow Cytometry in Bone Marrow and Apheresis Products for Assessment and Outcome Prediction in Multiple Myeloma Patients
Source: Front Oncol. 2021 Aug 13;11:708231. doi: 10.3389/fonc.2021.708231 (PMC8414971; doi:10.3389/fonc.2021.708231)

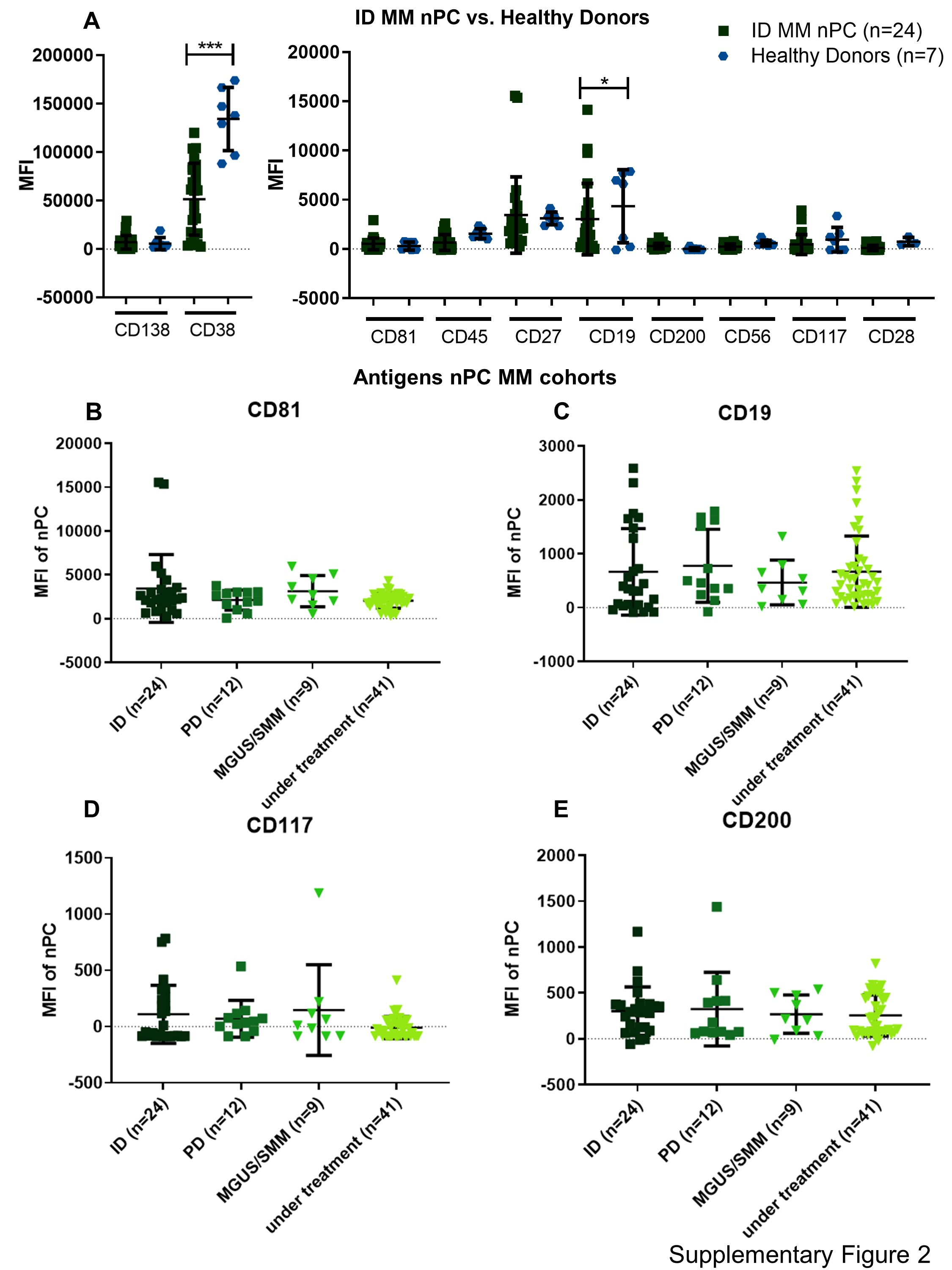

Supplement: Supplementary file 1 [file DataSheet_1.zip › Supp. Fig 2.JPG]

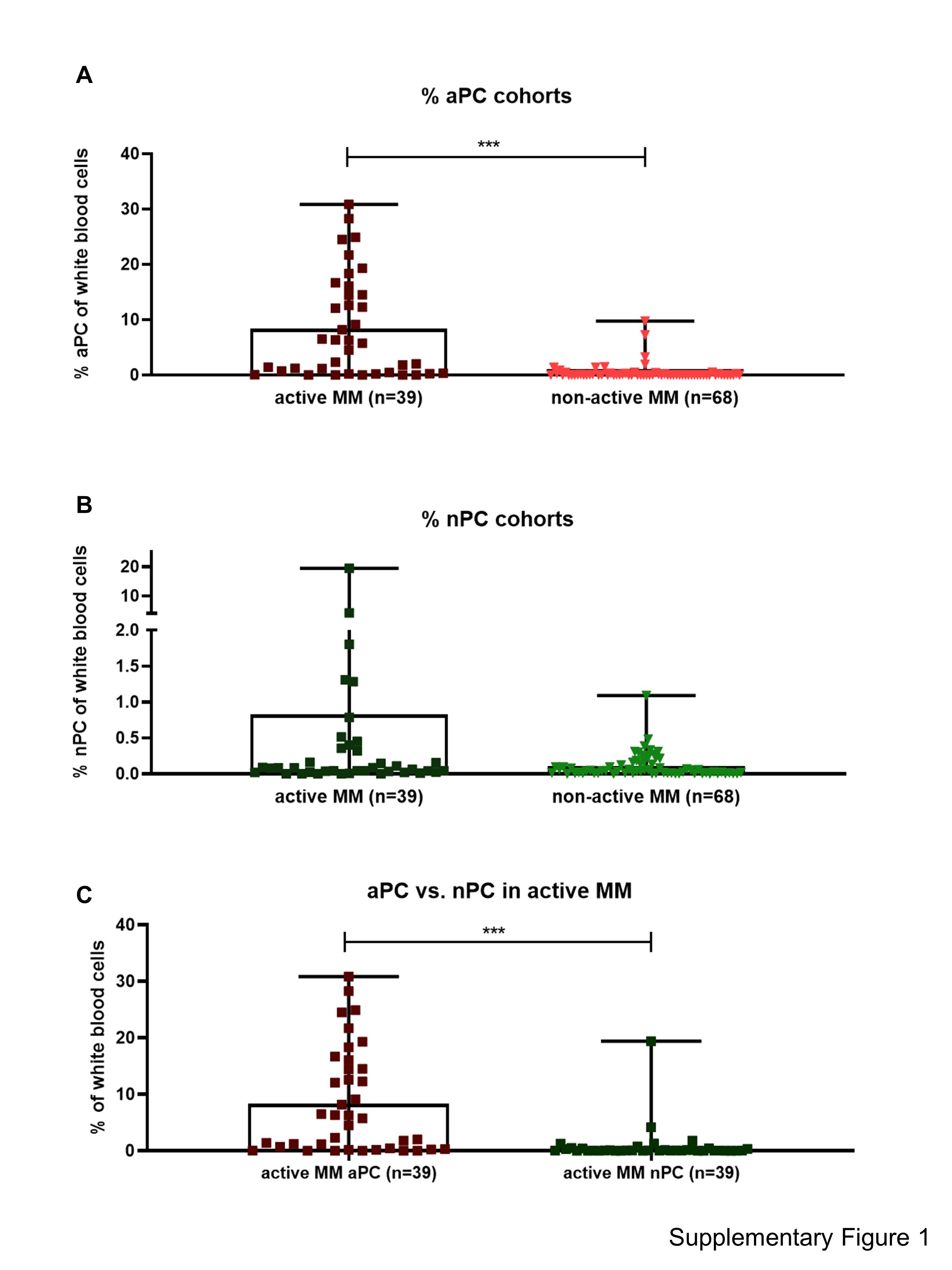

Supplement: Supplementary file 1 [file DataSheet_1.zip › Supp.Fig 1.JPG]

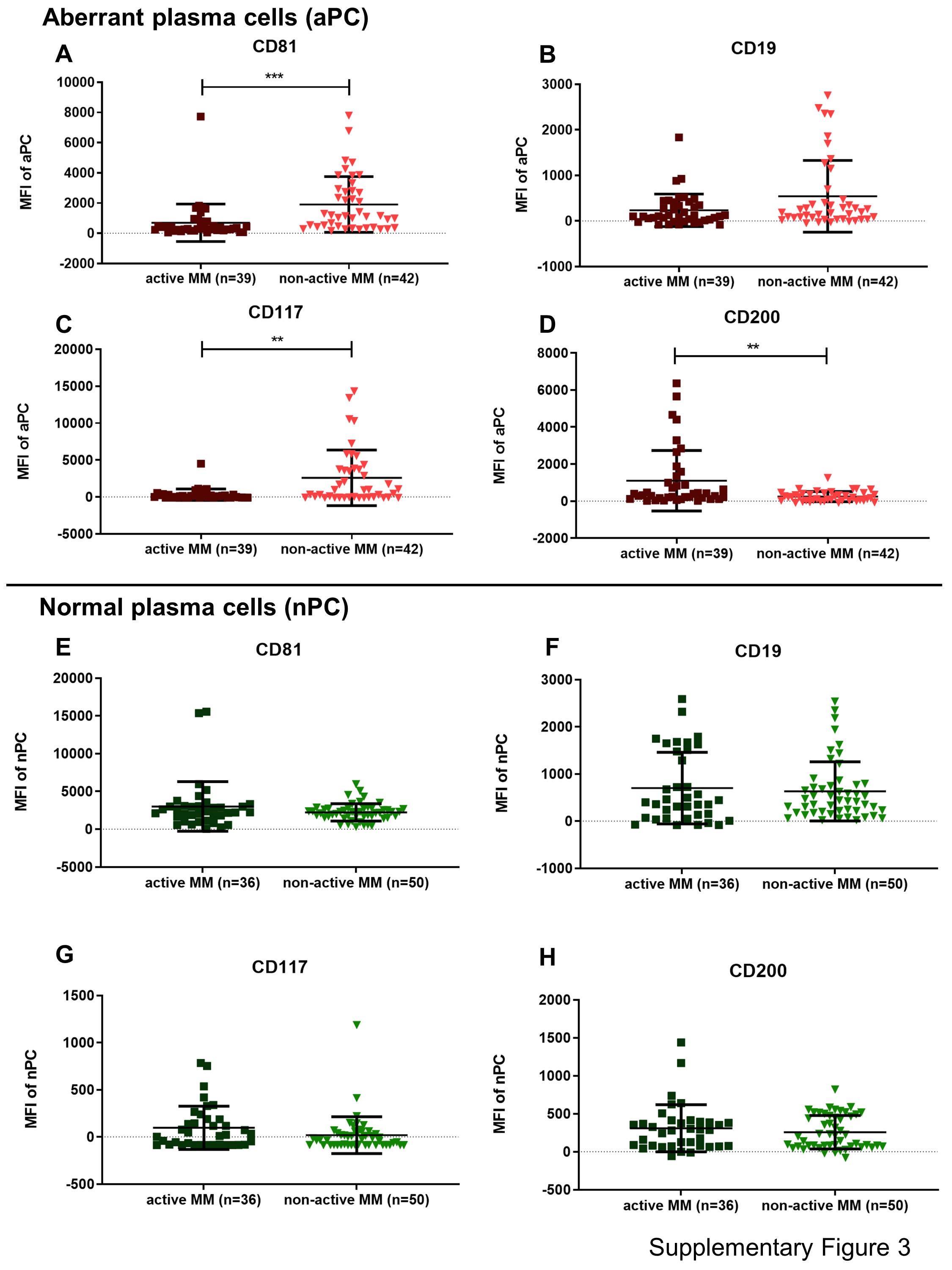

Supplement: Supplementary file 1 [file DataSheet_1.zip › Supp.Fig 3.JPG]
